# Signature methods for brain-computer interfaces

**DOI:** 10.1038/s41598-023-41326-8

**Published:** 2023-12-04

**Authors:** Xiaoqi Xu, Darrick Lee, Nicolas Drougard, Raphaëlle N. Roy

**Affiliations:** 1 Cerco, CNRS, Université de Toulouse, Toulouse, France; 2 University of Oxford, Oxford, UK; 3 ISAE-SUPAERO, Université de Toulouse, Toulouse, France

**Keywords:** Bioinformatics, Electroencephalography - EEG, Biomedical engineering, Applied mathematics

## Abstract

Brain-computer interfaces (BCIs) allow direct communication between one’s central nervous system and a computer without any muscle movement hence by-passing the peripheral nervous system. They can restore disabled people’s ability to interact with their environment, e.g. communication and wheelchair control. However, to this day their performance is still hindered by the non-stationarity of electroencephalography (EEG) signals, as well as their susceptibility to noise from the users’ environment and from their own physiological activity. Moreover, a non-negligible amount of users struggle to use BCI systems based on motor imagery. In this paper, a new method based on the path signature is introduced to tackle this problem by using features which are different from the usual power-based ones. The path signature is a series of iterated integrals computed from a multidimensional path. It is invariant under translation and time reparametrization, which makes it a robust feature for multichannel EEG time series. The performance can be further boosted by combining the path signature with the gold standard Riemannian classifier in the BCI field exploiting the geometric structure of symmetric positive definite (SPD) matrices. The results obtained on publicly available datasets show that the signature method is more robust to inter-user variability than classical ones, especially on noisy and low-quality data. Hence, this study paves the way towards the use of mathematical tools that until now have been neglected, in order to tackle the EEG-based BCI variability issue. It also sheds light on the lead-lag relationship captured by path signature which seems relevant to assess the underlying neural mechanisms.

## Introduction

A brain-computer interface (BCI) is a system that allows for interaction with machines using only brain activity and no muscular activity. The major motivation of early BCIs was restoring the ability of severely paralyzed people to communicate and interact with the environment^1^. A BCI is composed of several parts^2^: the signal acquisition system records, amplifies and digitizes brain signals; the preprocessing steps consist of removing noise and artifacts in order to improve the signal-to-noise ratio^3^; and the feature extraction and classification steps to finally transform brain signals into labels sent to the computer as information for explicit or implicit control.

There are many types of BCIs according to the brain signal used and the way to use the system. In this paper we focus on electroencephalography (EEG)-based BCIs using sensorimotor rhythms (SMR). EEG records the extracellular field potentials associated with neural activity with electrodes placed over the scalp. It is non-invasive, accessible, and has a high temporal resolution. It has been found^4–6^ that motor movement as well as motor imagery (MI, i.e. imagination of movement without actually moving) cause modulation in SMR manifested as a decrease of power in the alpha (8–13 Hz)/beta(13–30 Hz) frequency bands, known as event-related desynchronization (ERD), followed by an increase in the beta band, also known as beta rebound or event-related synchronization (ERS), after the actual or imagined movement. Movement or MI of different body parts is associated with an SMR modulation of different regions of the sensorimotor cortex, which leads to discriminant brain signals that allows the control of MI BCI.

One major obstacle to bring EEG-based BCI into everyday life for ordinary people is its lack of robustness due to the high variability of EEG signals. The distribution of EEG data changes between sessions and users which makes it difficult to establish a robust classifier that works across time and users. To deal with this problem, most studies have focused on applying transfer learning techniques to align the data distributions^7,8^. However, there are some less well known tools that enjoy interesting properties to tackle the variability problem, for example the path signature of a time series is translation invariant and independent of time parametrization. So the path signature-based features may be more robust to the variance related to change of power and speed. This is the initial motivation of applying path signature to BCI applications.

The path signature was originally introduced by Chen^9^ in the framework of piece-wise smooth curves, then developed by Lyons et al.^10^ to study stochastic differential equations. Recently, it has been used as a feature generation method for time series in various applications such as handwritten character recognition^11^, diagnosis of bipolar disorder and borderline personality disorder based on daily mood ratings^12^, and diagnosis of Alzheimer’s disease based on hippocampal and brain volume time series^13^, just to name a few. To our knowledge, to this day this method has never been applied to BCI applications.

In this study, we propose two signature-based methods for EEG-based BCI applications to tackle the user-variability issue. The main tools are detailed in the next section, then the experiments performed on several publicly available datasets and their results are presented, followed by a discussion of the results and further analyses.

## Methods

The main tools used to compute the signature for EEG-based BCI applications are given in this section. To go further, interested readers can find more details of the theory and also the practical use cases of path signature in the tutorial written by Chevyrev and Kormilitzin^14^.

### Path signature

Suppose we have a *d*-dimensional multivariate time series $X_t = (X^1_t, \ldots , X^d_t)$, where $X^i : [a,b] \rightarrow {\mathbb {R}}$ is a component of this continuous time series. We can interpret this multivariate time series as a continuous path $X_t: [a, b] \rightarrow {\mathbb {R}}^d$ from [*a*, *b*] to ${\mathbb {R}}^d$. The *path signature* of $X_t$ is an infinite sequence of tensors (arrays) of increasing dimension,

$$\begin{aligned} S(X)_{a,b} = (1, S_1(X)_{a,b}, S_2(X)_{a,b}, \ldots ), \end{aligned}$$

where $S_k(X)_{a,b} \in {\mathbb {R}}^{d^k}$ is a *k*-dimensional array of numbers of length *d* in each dimension, which we call the *level k path signature of X*. A component of the array $S_k(X)_{a,b}$ is indexed by a *multi-index* $I = (i_1, \ldots , i_k)$, where each $i_j \in \{1, \ldots , d\}$. The *I*-coordinate of $S_k(X)_{a,b}$ is defined by the iterated integral

$$\begin{aligned} S_k^I(X)_{a,b} = \int \limits _{a< t_k< b} \int \limits _{a< t_{k-1}< t_k} \ldots \int \limits _{a< t_1 < t_2} {\dot{X}}^{i_1}_{t_1} {\dot{X}}^{i_2}_{t_2} \ldots {\dot{X}}^{i_k}_{t_k} \,dt_1 dt_2 \ldots dt_k. \end{aligned}$$

In the following we will use the terms path signature or signature interchangeably. Furthermore, we omit the subscript for the endpoints (*a*, *b*) when the signature is taken over the entire domain of the path, $S(X) {:=}S(X)_{a,b}$.

Here we explicitly describe the first two levels of the signature to gain a more intuitive understanding. For $i \in \{1, \ldots , d\}$, the *i*-component of the first level signature $S_1(X)$ is

$$\begin{aligned} S_1^i(X) = \int \limits _{a< t< b} {\dot{X}}^i_{t} dt = X^i_b - X^i_a, \end{aligned}$$

the displacement of the path in the *i* component (i.e. the difference between the ending and starting point in dimension *i*).

The second level signature captures richer information about the relationship between the pair of path components and it has a beautiful geometric interpretation. Suppose $i, j \in \{1, \ldots , d\}$. As in the first level, we can perform the explicit computation to obtain

$$\begin{aligned} S^{i,j}_{2}(X) = \int \limits _{a< t_2< b} \int \limits _{a< t_1< t_2} {\dot{X}}^{i}_{t_1} {\dot{X}}^{j}_{t_2} \, dt_1 dt_2 = \int \limits _{a< t_2 < b} (X^i_{t_2} - X^i_{a}) {\dot{X}}^{j}_{t_2} dt_2. \end{aligned}$$

**Figure 1:**
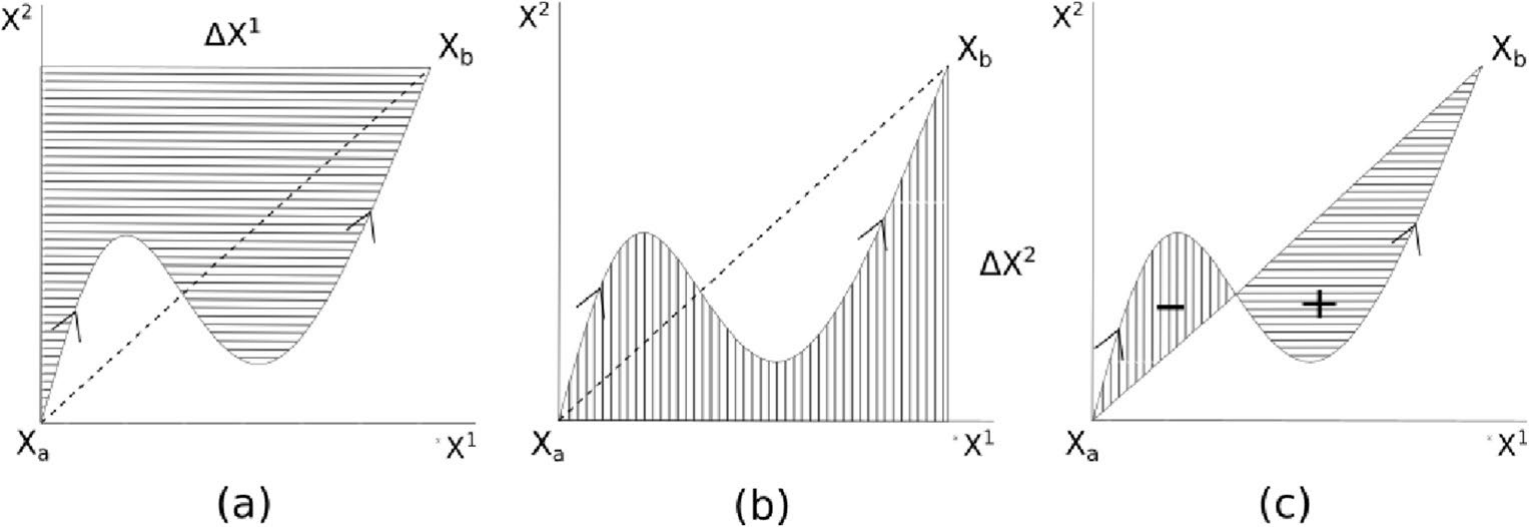
Geometric interpretation of level 2 signature. The shaded area in the subfigures represents (**a**) $S(X)^{1, 2}_{a, b}$, (**b**) $S(X)^{2, 1}_{a, b}$, and (**c**) the signed area (thedifference between areas “$+$” and “−”) respectively.

This quantity can be interpreted as the area bounded by the path $X_{[a, b]}$ and the $X^{j}$-axis, as shown in Fig. 1a and b. From this, we can see that the signature is in general not symmetric with respect to its components; for instance $S^{i,j}_2(X) \ne S^{j,i}_2(X)$. In fact, the difference

$$\begin{aligned} \frac{1}{2} \left( S^{i,j}_2(X) - S^{j,i}_2(X)\right) \end{aligned}$$

is the *signed area* bounded by the path *X* (appended with a linear path from the end point $X_b$ to the initial point $X_a$, see Fig. 1c).

While higher levels of the signature are more difficult to interpret geometrically, we can see from these two examples that the signature captures geometric features of the path. In fact, the path signature fully characterizes the path up to an equivalence relation called tree-like equivalence (roughly speaking, two paths are tree-like equivalent if they differ by a finite number of retracings)^15^. Even more crucial for data science applications, finite linear combinations of signature terms,

$$\begin{aligned} \sum _{j=1}^n a_j S^{I_j}(X), \end{aligned}$$

where $a_j \in {\mathbb {R}}$, and each $I_j$ is a multi-index (possibly of different lengths), can approximate nonlinear functions on the space of paths under suitable conditions^16^. This property allows us to transform a non-linear classification problem on the space of paths into a linear classification problem on the space of signature features.

The application of path signatures for EEG-based BCI is strongly motivated by its invariance properties.

1. *(Translation Invariance.)* For any path $X_t : [a,b] \rightarrow {\mathbb {R}}^d$ and any vector $v \in {\mathbb {R}}^d$, we have $S(X)_{a,b} = S(X+v)_{a,b}$.
2. *(Reparametrization Invariance.)* For any path $X_t: [a,b] \rightarrow {\mathbb {R}}^d$ and any reparametrization $\phi : [a,b] \rightarrow [c,d]$, which is a monotone increasing, bijective function, we have $S(X)_{a,b} = S(X \circ \phi )_{c,d}$.

In practice, the path signature is truncated at a certain level so it provides a fixed length feature vector of the time series regardless of the number of time steps. The path signature has further algebraic properties which leads to effective algorithms in an online context. Suppose $X_t: [a,b] \rightarrow {\mathbb {R}}^d$ is signal for which we have already computed the path signature, and $Y_t: [b,c] \rightarrow {\mathbb {R}}^d$ is a newly obtained signal. We can compute the signature of the concatenated path $(X*Y)_t: [a,c] \rightarrow {\mathbb {R}}^d$ by taking the tensor product of the two signatures, $S(X*Y)_{a,c} = S(X)_{a,b} \otimes S(Y)_{b,c}$; this relation is called *Chen’s identity*^14^. In practice, $Y_t$ would consist of one additional time point, and by using the fused multiply-exponentiate algorithm^17^, the complexity of computing $S_{\le k}(X*Y)$ given $S_{\le k}(X)$ is $O(d^k)$, where *k* is the truncation level of the signature.

Indeed, as mentioned earlier, EEG data vary greatly between sessions and users. By using the path signature, we expect some of these variations (e.g. the covariate shift or the temporal difference) to be absent for the features using this method, and would therefore allow us to build more robust BCIs.

### Cyclicity analysis

The cyclic structure or lead-lag relationship (the temporal ordering of cyclic signals) of a multidimensional path can be recovered from the second level signature and has been applied successfully to analyse fMRI data^18–20^. We define the *lead matrix*^18^ $L \in {\mathbb {R}}^{d \times d}$ by

$$\begin{aligned} L(X) {:=}\frac{1}{2}\left( S_2(X) - S_2(X)^\intercal \right) , \end{aligned}$$

where $S_2(X) \in {\mathbb {R}}^{d \times d}$ is the second level signature as a $d \times d$ matrix, and $S_2(X)^\intercal$ is its transpose. In particular, the (*i*, *j*) entry of *L* is

$$\begin{aligned} L_{i,j}(X) = \frac{1}{2}\left( S_2^{i,j}(X) - S_2^{j,i}(X)\right) , \end{aligned}$$

the signed area of the path *X* projected onto the coordinates $(X^i_t, X^j_t)$, as discussed in the previous section. An observation from Baryshnikov and Schlafly^18^ is that a positive value of $L_{i,j}(X)$ (or equivalently, a negative value of $L_{j,i}(X)$ due to the skew-symmetry of *L*(*X*)) can be interpreted as an indicator that the signal $X^i$ is *leading* the signal $X^j$.

In fact, we can gain further insight into the temporal ordering of cyclic signals from the lead matrix. We consider a simple example to demonstrate this fact. Consider the time series $X_t : [0,T] \rightarrow {\mathbb {R}}^n$, where $X^i_t {:=}\sin (t - \alpha _i)$, where $\alpha _i$ denotes a phase shift.

The goal is to recover the cyclic order of the components, or equivalently the system of offsets $\alpha _i$, from the lead matrix. The lead matrix of $X_t$ can be explicitly computed, where

$$\begin{aligned} L_{i,j}(X) = \frac{1}{2} \int \limits _0^T X^i_t {\dot{X}}^j_t - {\dot{X}}^i_t X^j_t \, dt =\frac{1}{2} \int \limits _0^T \sin (t-\alpha _i) \cos (t-\alpha _j) - \cos (t-\alpha _i) \sin (t-\alpha _j) \, dt = \frac{T}{2} \sin (\alpha _i - \alpha _j). \end{aligned}$$

**Figure 2:**
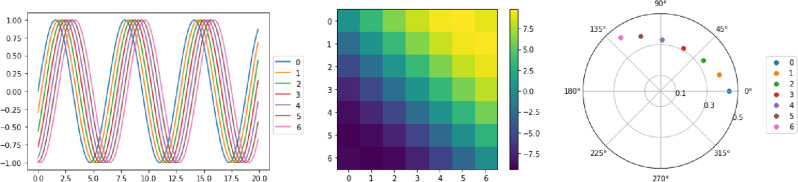
The signals are $\sin (t - \alpha _i)$ shown at the left. In the middle is the lead matrix in which yellow corresponds to positive values and blue to negative values. If $L_{i,j}$ is positive it means signal *i* is leading signal *j*. At the right is the first eigenvector of the lead matrix. We see a perfect recovery of the cyclic order from the phase of the elements of the first pair of eigenvectors.

We can rewrite $L = \frac{T}{2}( x y^\intercal - y x^\intercal )$, where $x = (\sin \alpha _1, \ldots , \sin \alpha _n)^\intercal$ and $y = (\cos \alpha _1, \ldots , \cos \alpha _n)^\intercal$; in particular, it is a rank 2 matrix. Following the analysis in Baryshnikov and Schlafly^18^, the eigenvectors corresponding to the nonzero eigenvalues are

$$\begin{aligned} v_1 = e^{i \psi } ( e^{2 \pi i \alpha _1}, \ldots , e^{2\pi i \alpha _n}), \quad v_2 = {\bar{v}}_1, \end{aligned}$$

where $\psi$ is a phase, and ${\bar{v}}_1$ denotes the complex conjugate of $v_1$. Thus, we find that the phase of the complex components in the eigenvector $v_1$ recovers the temporal ordering of the signals, and we show an numerical illustration of this example in Fig. 2.

If the path *X* contains more than one set of lagged sine waves, there will be the same number of non zero conjugate pairs of eigenvalues as the number of sets of sine waves. The cyclic order of each set of sine waves can still be recovered from eigenvectors. The magnitude of the eigenvalues indicates the magnitude of the sine wave and the magnitude of the elements in each eigenvector indicates the correlation with the corresponding sine wave^21^.

**Figure 3:**
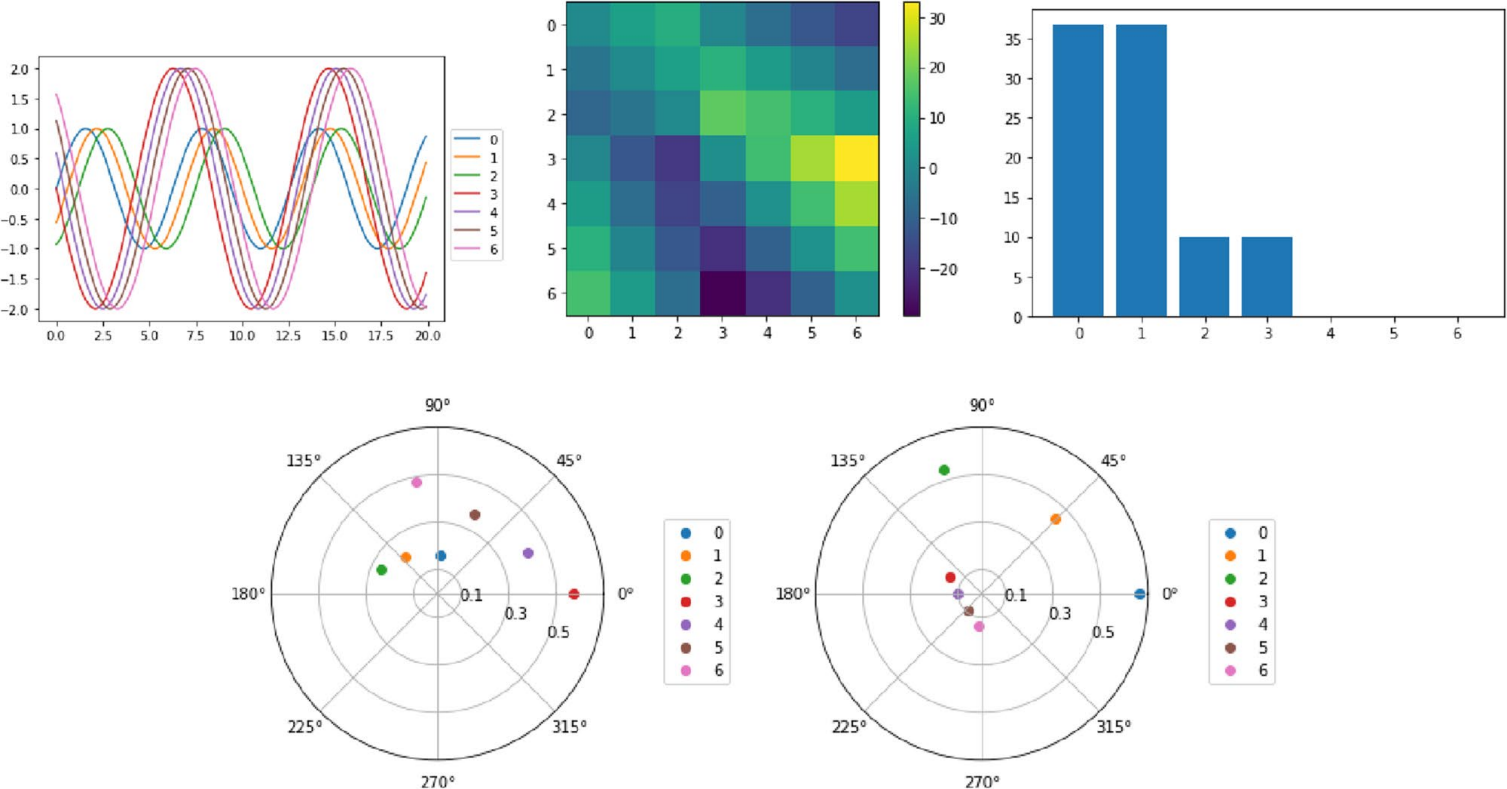
First row: on the left is the plot of 2 sets of sin waves; in the middle is the corresponding lead matrix; on the right is the absolute value of the eigenvalues of the lead matrix. Second row: the 2 eigenvectors corresponding to the 2 pairs of non zero eigenvalues.

Figure 3 shows an example with 2 sets of sine waves. There are 2 conjugate pairs of eigenvalues and 2 blocks in the lead matrix. With the eigenvectors corresponding to the 2 non zero eigenvalues, the cyclic order is again completely recovered. While we only considered simple sinusoids in this example, we emphasize that the same results would hold for any reparametrization of the sine waves, due to reparametrization invariance.

### Riemannian classifier

The Riemannian classifier is the gold standard classifier in the field of brain-computer interfaces^7^. It takes as features the covariance matrices of multi-channel EEG data. When estimated with enough samples, covariance matrices are symmetric positive definite (SPD) matrices, i.e. they belong to the set $\{A \,|\, A^\intercal = A, \lambda _i > 0, \,\forall i\}$, where $\lambda _i$ are eigenvalues of *A*. This set is an open subset of ${\mathbb {R}}^{n\times n}$, so it is a manifold. However, geodesics induced by the Euclidean embedding may leave the SPD manifold. To remedy this, an intrinsic Riemannian metric of the manifold has to be used. The most popular solution in the medical imaging domain is the affine-invariant metric^22^, and the Log-Euclidean metrics^23^ provides a fast approximation when the data are concentrated with respect to the curvature. The following study used pyRiemann^24^ package for the implementation of the Riemannian classifier, and only the affine-invariant metric is used.

The Riemannian classifier has its name since it uses Riemannian geometry to compute the distance between covariance matrices, but the core of the classification algorithm could be any classical machine learning algorithm. For example, with the minimum distance to mean (MDM) classifier, it computes the distance between a sample and the mean of each class and labels it with the label of the nearest class. The novelty is due to the fact that the distance and mean are computed in the Riemannian sense. Another variant is to project all covariance matrices to the tangent plan of a reference point (e.g. identity matrix, geometric mean) via the logarithmic map and then apply classical classifiers such as support vector machine (SVM) in the Euclidean tangent space^25^.

### Datasets

In accordance with the principles of open science, we have exclusively worked with publicly available datasets. We have further chosen the most used datasets among open EEG-based BCI datasets for MI BCI applications: the BCI competition IV 2a dataset^26^ and the Physionet motor imagery dataset^27^.

The BCI competition IV 2a dataset is an indispensable classical MI BCI dataset which consists of data collected from 9 subjects on 2 sessions on different days. Each session contains a total of 288 trials of 4 tasks: imagination of movement of left hand, right hand, feet and tongue. Each trial lasts for 6 s with 2 s of resting state at the beginning. After the cue appears on the screen at $t=2$s, participants have to perform the motor imagery task. EEG signals were recorded using 22 Ag/AgCl electrodes placed according to the international 10–20 system with the left mastoid as reference and right mastoid as ground electrode. Signals were down-sampled to 250 Hz and band-pass filtered into the 0.5–100 Hz band. A 50 Hz notch filter was applied. There were also 3 EOG channels, but they were not used in our experiments. Trials were checked by human experts and those containing artifacts were marked by the dataset providers. The trials marked with artifacts were excluded in our study.

The Physionet MI dataset contains data recorded from 109 subjects performing motor movement and imagery, but only motor imagery data were used in our experiments. There is only 1 session consisting of 14 runs, each including 2 trials of 1 min resting state (eyes open/closed), and 3 runs of 2 min with the following 4 tasks: open and close left or right fist, imagine opening and closing left or right fist, open and close both fists or both feet, imagine opening and closing both fists or both feet. EEG signals were recorded using the BCI2000 system with 64 channels placed according to the international 10–20 system and were down-sampled to 160 Hz. The data of 4 subjects were rejected because they do not have the same number of trials and time steps as other subjects.

## Results and discussion

**Figure 4:**
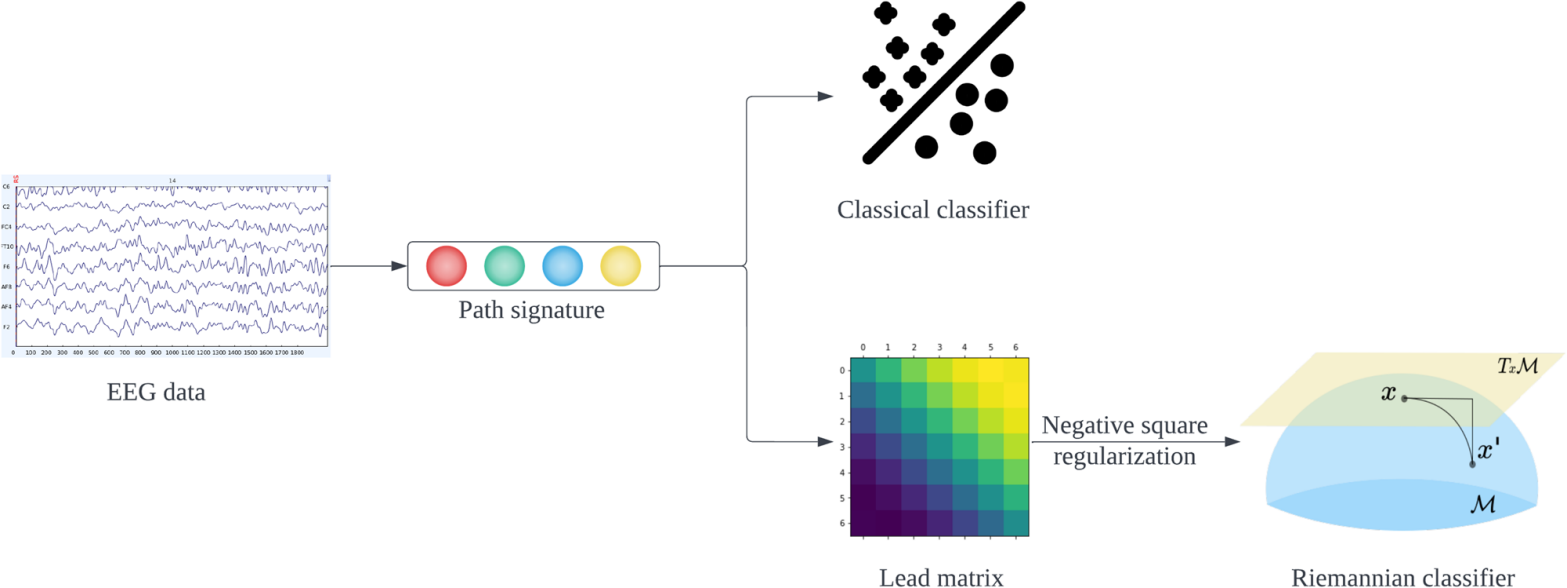
The first study uses the path signature directly as a feature vector. The second study takes the negative square of the lead matrix constructed from the second level signature and adds a regularization term to get a symmetric positive definite (SPD) matrix to be used as features with the Riemannian classifier.

In this section two ways of applying the path signature on EEG-based BCIs, as illustrated in Fig. 4, are presented, followed by a cyclicity analysis on EEG data. We use the Signatory^17^ Python package for signature computations.

### First study

In the first study, the use of the path signature as a feature map was explored. More precisely, we considered the EEG signal $X_t$ as an *n*-dimensional path $X: [0, T] \mapsto {\mathbb {R}}^n$ and computed the truncated signature up to level *k*. Then a classical classifier was applied on this feature vector. The focus here was exploring the performance with different truncation levels and classifiers, so only the most classical dataset, the BCI competition IV 2a dataset, was used.

**Figure 5:**
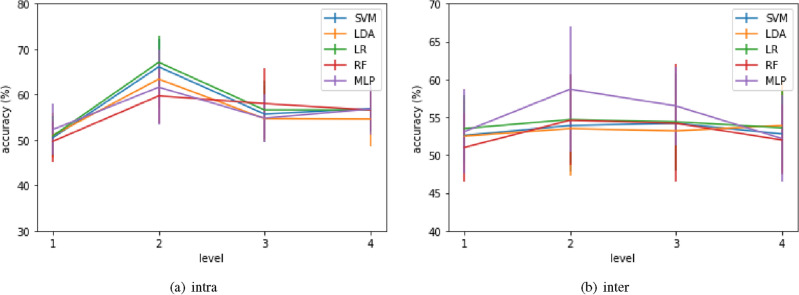
Classification accuracy of standard classifiers using different truncation levels (x-axis) of path signature on BCI Competition IV 2a dataset (left vs. right).

Cross-validation (Ten-fold for intra-subject and leave-one-out for inter-subject) was performed on the data with the left versus right motor-imagery paradigm. The results are summarized in Table 1 and Fig. 5. The full name of the classifiers associated with the acronyms used in the table are: support vector machine (SVM), linear discriminant analysis (LDA), logistic regression (LR), random forest (RF), and multilayer perceptron (MLP). Note that even though we call it “raw data”, the data was still filtered into the 8–30 Hz frequency band relevant to motor imagery before computing path signature.

In both intra- and inter-subject classification, the best results were achieved by level 2 signature: 67.1% with LR for intra-subject classification and 58.7% with MLP for inter-subject classification. As discussed in the Methods section, the second level signature captures the cyclic order and can be reformulated by lead matrices. By focusing our analysis on the second level, we can employ the matrix structure, leading to the second study of this paper.

**Table 1:** Mean cross-validation score (standard deviation in parentheses) of various classifiers and truncation levels of path signature applied on raw data of the BCI competition IV 2a dataset (left vs. right).

level	1	2	3	4
Intra				
SVM	50.5(12.9)	66.1(15.2)	55.7(13.3)	56.9(13.9)
LDA	51.0(12.6)	63.4(14.0)	54.7(14.2)	54.6(14.2)
LR	50.9(11.3)	**67.1**(13.5)	56.6(13.3)	56.6(14.0)
RF	49.7(11.6)	59.7(16.1)	58.0(16.8)	56.6(18.0)
MLP	52.3(11.2)	61.6(16.4)	54.8(13.3)	56.8(15.1)
Inter				
SVM	52.6(3.6)	53.9(6.1)	54.2(6.2)	52.8(4.9)
LDA	52.5(4.9)	53.5(6.2)	53.2(4.9)	53.9(6.0)
LR	53.5(4.4)	54.7(5.8)	54.4(6.4)	53.6(4.9)
RF	51.0(4.5)	54.6(6.0)	54.2(7.8)	52.0(4.6)
MLP	53.1(5.6)	**58.7**(8.3)	56.5(5.2)	52.2(5.7)

The values in bold are the best performances in intra-subject and inter-subject classification respectively.

### Second study

It is clear that the lead matrix *L* is skew-symmetric, i.e. $L^\intercal = -L$. We can turn this into a symmetric positive semi-definite matrix by taking the negative square, as $A = -L^2 = L^\intercal L$. In fact, let *v* be an arbitrary vector, we have $v^\intercal A v = v^\intercal L^\intercal L v = \Vert L v \Vert ^2 \ge 0$.

The matrix *A* can be further turned into an SPD matrix by adding a small value on its diagonal^28^. Now that we have the SPD matrix $A + \varepsilon I$, we can use them as features and leverage the advantages of Riemannian classifiers. This idea is tested here on both publicly available BCI datasets, both for intra- and inter-subject classification to further evaluate the usefulness of signature features, including a benchmark with usual covariance features. The results are summarized in Table 2 ($\varepsilon$ was set to 0.001).

**Table 2:** Intra- and inter-subject cross-validation accuracy (standard deviation between parentheses) using Riemannian tangent space classifier on signature based SPD matrices and covariance matrices respectively on two open MI-BCI datasets.

	Signature		Covariance	
	Intra	Inter	Intra	Inter
BCI competition IV 2a	71.4(18.1)	66.1(11.8)	81.1(16.6)	69.2(15.9)
Physionet MI-BCI	60.1(23.9)	47.0(11.0)	63.8(24.2)	46.2(14.8)

Although the signature-based method falls behind traditional Riemannian method for intra-subject classification, the difference is not substantial for inter-subject classification, and it shows better robustness, especially on Physionet dataset with a much larger number of subjects and a relatively low mean classification accuracy with the state-of-the-art methods. Not only is the mean accuracy slightly better (47.0% vs. 46.2%), the standard deviation is also smaller: 11.0% for signature based method and 14.8% for the traditional Riemannian classifier.

**Figure 6:**
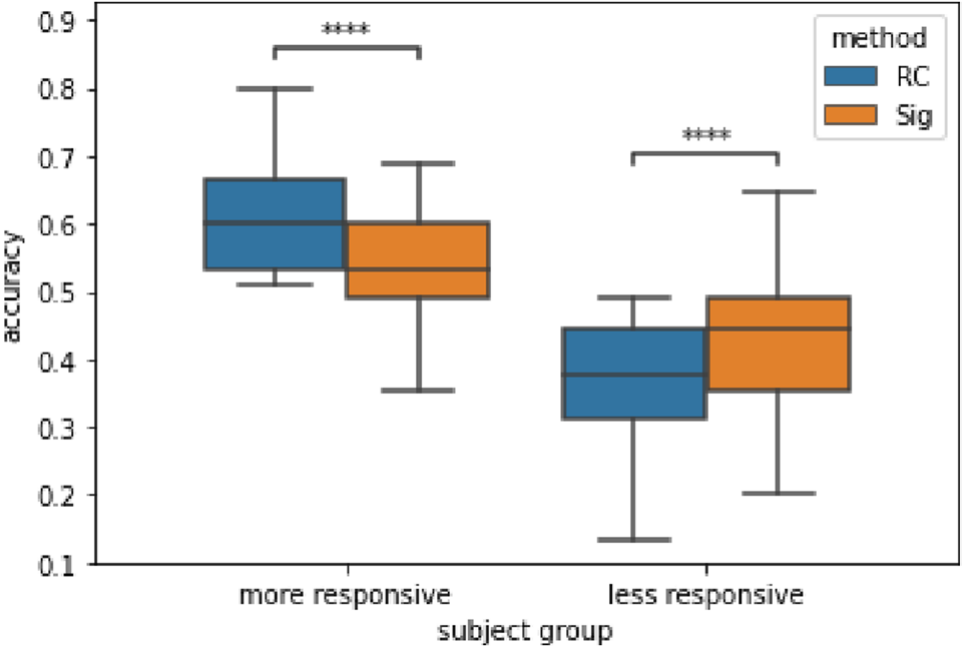
Inter-subject classification accuracy (grouped by performance under RC with a 50% threshold) on the Physionet dataset with leave-one-out cross-validation. A Riemannian classifier is applied on covariance matrices (RC) and on signature-based SPD matrices (Sig) respectively. The *p*-values of the paired t-test with Bonferroni correction applied on accuracy of RC and Sig for good and bad groups are $6.828e-05$ and $8.351e-06$ respectively (marked with **** in the figure).

If we divide subjects into more and less responsive subjects according to their score under traditional Riemannian classifier with a 50% threshold (chance level^29^), we were able to see the clear advantage of the signature based method for subjects with low classification accuracy from Fig. 6. Note that 67 of the 105 subjects are categorized as less responsive.

### Cyclicity analysis on EEG data

**Figure 7:**
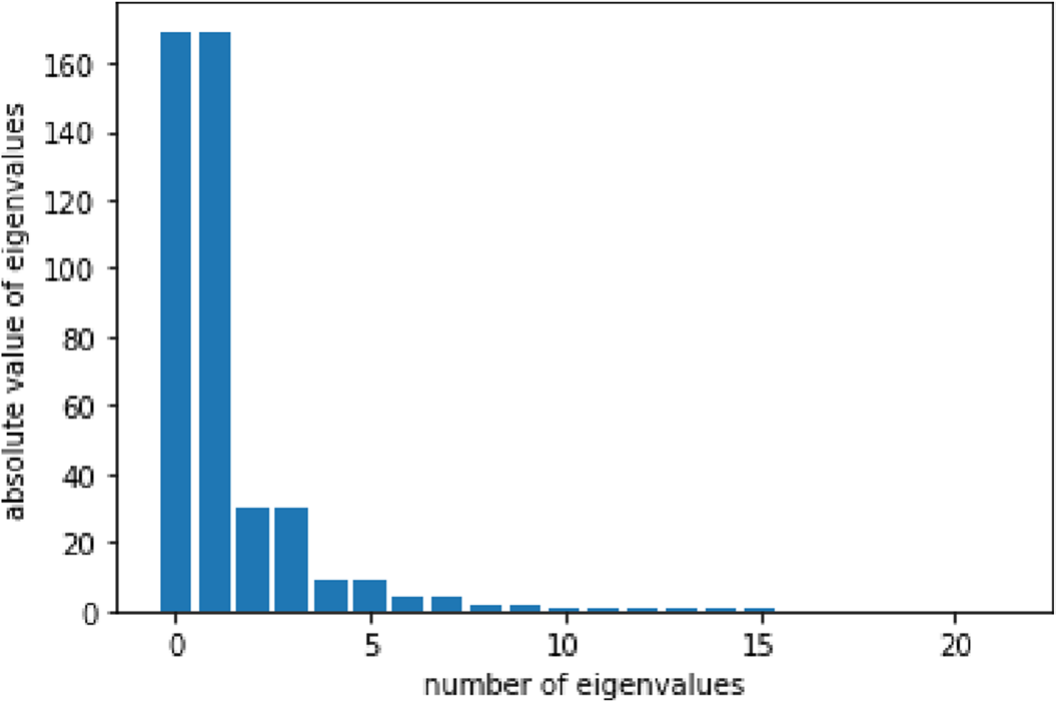
Absolute value of the eigenvalues of the lead matrix averaged across trials.

To better understand the stronger performance of the second level signature, a cyclicity analysis as described in the Methods section was conducted on the same EEG data used in the first study (BCI Competition IV 2a dataset). The eigenvalues and eigenvectors of the lead matrices were computed. Figure 7 shows the absolute value of the eigenvalues averaged across trials and participants. The first 2 conjugate pairs of eigenvalues dominate, which guarantees a good power of explicability of the corresponding eigenvectors.

**Figure 8:**
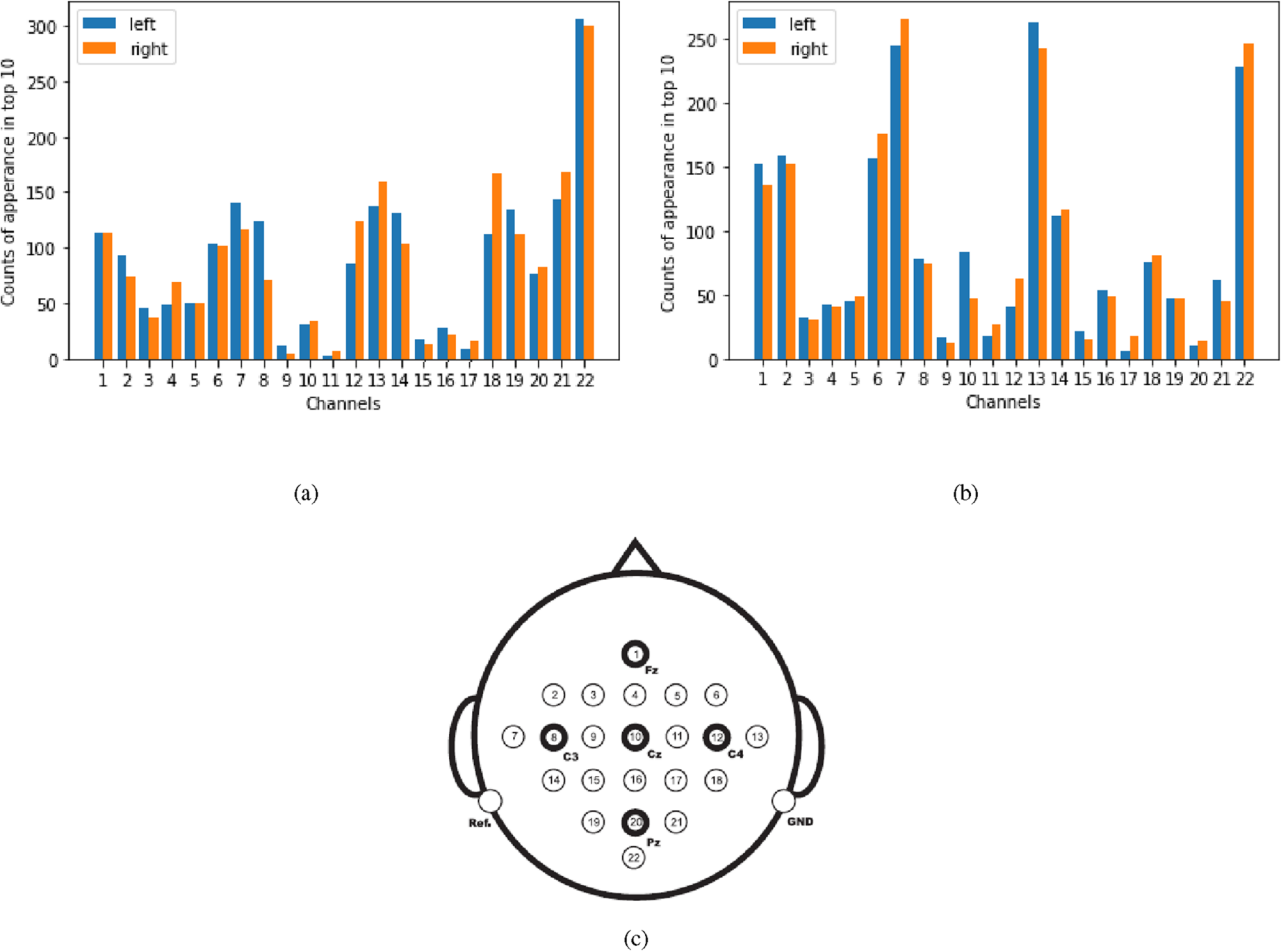
Frequency that a channel appears in the top 10 largest elements of the first (**a**) and second (**b**) leading eigenvectors of the lead matrix in the left/right MI conditions separately (over all subjects in the BCI Competition IV 2a dataset). The channel locations^26^ are shown in (**c**).

Figure 8 shows the number of times that one channel appeared in the top 10 largest elements of the first 2 eigenvectors corresponding to the 2 largest pairs of eigenvalues. The color of the bars indicates left/right MI. For the first eigenvector, the channel POz (labeled as 22 in the dataset) from the parieto-occipital region had the largest counts. This might be due to the fact that during a task, brain signals are dominated by visual related signal processed in the occipital lobe and attentional networks related signals from the parietal lobe. Note that there was little difference between left/right conditions. For the second eigenvector, there was a large contribution from channels located above the sensorimotor cortex (e.g. C5 and C6 or 7 and 13 according to the channel labels in the dataset) and a clear difference between left/right conditions.

## Conclusion

This paper explored applications of the path signature for EEG-based BCIs. The first study used the path signature directly as a feature vector. Promising results were achieved with the signature truncated at the second level. The second study used the negative square of the lead matrices constructed from the second level signature and added a regularization term to obtain SPD matrices as features. A Riemannian classifier was applied on these signature-based SPD matrices. The classification results on several publicly available MI BCI datasets were compared to those with a Riemannian classifier applied on covariance matrices. The signature-based method showed better performance and robustness on users where the traditional use of Riemannian classifier fails although remaining close to the chance level.

Even though lead-lag relationships in EEG signals could be established by estimating phase differences via a traditional time-frequency analysis, the benefit of using the signature is that it does not rely on the assumption of periodicity, which is more realistic. Moreover, there is no need to first filter the signal into a narrow frequency band to get an interpretable phase. The lead matrix contains the phase information of oscillations of different frequencies.

Moreover, there is evidence that the lead-lag relationship, i.e. the ordering information, does encode information and have a functional role in the brain. At the macroscopic level, cortical travelling waves (oscillations with systematic phase offsets) have been observed in the EEG recording at a wide range of brain areas, e.g. motor cortex, visual cortex and hippocampus^30^. In particular, the travelling waves in the motor cortex are suggested to have the functional role of movement preparation and motor coordination^31^, which may justify the relevance to the motor imagery based BCI. At the microscopic level, the neural sequence (the sequence of neuronal activity in which neurons are transiently active during task trials with different neurons active at different parts of the trial) has been used as the representation for a sensorimotor task of a mouse navigating a T-maze^32^. Zhou et al. argued that the neural sequences represent an ideal and flexible dynamical regime for the brain to read out time information^33^. However the link between mental states and the lead-lag relationship between channels needs to be further investigated and validated. The lead-lag relationship should also be interpreted with caveat since the implication of lead/lag based on the sign of a signed area assumes that the signals are consubstantial. This is not the case with EEG signal which might have opposite trends in different channels.

Hence, this article advocated for the use of a mathematical method that was until now unexplored for both EEG analysis and BCI applications. Promising results are found which open new perspectives on how to design BCIs that would be more robust to inter-subject variability and that might help tackle – at least in part– the so-called BCI illiteracy. The path signature provides a novel way to generate features from multi-channel EEG data. The features are invariant under time reparametrization and translation and they capture the lead-lag relationship between channels. In the future, a thorough comparison with more methods and on more open datasets via MOABB^34^ need to be done to further validate the utility of path signature for BCI applications. Ensemble learning could be employed to combine the covariance-based features and the signature-based features to further boost classification accuracy, as it has been shown to be effective with functional connectivity^35^. Besides, some general techniques to improve the performance of the signature method^36^, such as the lead-lag augmentation of the times series, could be attempted. It would also be interesting to investigate the link between the lead-lag relationship and other connectivity measures.

## Data Availability

The datasets analysed during the current study are publicly available. The BCI competition IV 2a dataset^26^ can be downloaded from https://www.bbci.de/competition/iv/. The Physionet motor imagery dataset^27^ can be downloaded from https://physionet.org/content/eegmmidb/1.0.0/.

## Code availability

The code is available at https://github.com/XiaoqiXu77/Signature_BCI.
